# Production of Reduced Graphene Oxide by Using Three Different Microorganisms and Investigation of Their Cell Interactions

**DOI:** 10.1021/acsomega.3c03213

**Published:** 2023-08-18

**Authors:** Guldem Utkan, Gorkem Yumusak, Beste Cagdas Tunali, Tarik Ozturk, Mustafa Turk

**Affiliations:** † SUNUM Nanotechnology Research Center,Sabanci University, Istanbul 34956,Turkey; ‡ Department of Metallurgical and Materials Engineering, Faculty of Engineering, Marmara University, Istanbul 34722,Turkey; § Department of Bioengineering, Faculty of Engineering, Kirikkale University, Kirikkale 71450,Turkey; ∥ Food Institute, Marmara Research Center, TUBITAK, Kocaeli 41470,Turkey

## Abstract

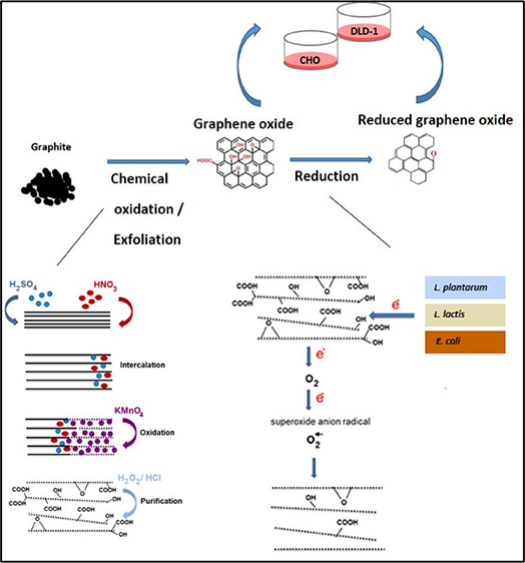

Despite the huge and efficient functionalities of reduced graphene oxide (RGO) for bioengineering applications, the use of harsh chemicals and unfavorable techniques in their production remains a major challenge. Microbial production of reduced graphene oxide (RGO) using specific bacterial strains has gained interest as a sustainable and efficient method. The reduction of GO to RGO by selected bacterial strains was achieved through their enzymatic activities and resulted in the removal of oxygen functional groups from GO, leading to the formation of RGO with enhanced structural integrity. The use of microorganisms offers a sustainable approach, utilizing renewable carbon sources and mild reaction conditions. This study investigates the production of RGO using three different bacterial strains: *Lactococcus lactis* (*L. Lactis*), *Lactobacillus plantarum* (*L. plantarum*), and *Escherichia coli* (*E. coli*) and evaluates its toxicity for safe utilization. The aim is to assess the quality of the produced RGO and evaluate its toxicity for potential applications. Thus, this study focused on the microbial production of reduced graphene oxides well as the investigation of their cellular interactions. Graphite-derived graphene oxide was used as a starting material and microbially reduced GO products were characterized using the FTIR, Raman, XRD, TGA, and XPS methods to determine their physical and chemical properties. FTIR shows that the epoxy and some of the alkoxy and carboxyl functional groups were reduced by *E. coli* and *L. lactis*, whereas the alkoxy groups were mostly reduced by *L. plantarum*. The *I*_D_/*I*_G_ ratio from Raman spectra was found as 2.41 for GO. A substantial decrease in the ratio as well as defects was observed as 1.26, 1.35, and 1.46 for ERGO, LLRGO, and LPRGO after microbial reduction. The XRD analysis also showed a significant reduction in the interlayer spacing of the GO from 0.89 to 0.34 nm for all the reduced graphene oxides. TGA results showed that reduction of GO with *L. lactis* provided more reduction than other bacteria and formed a structure closer to graphene. Similarly, analysis with XPS showed that L lactis provides the most effective reduction with a C/O ratio of 3.70. In the XPS results obtained with all bacteria, it was observed that the C/O ratio increased because of the microbial reduction. Toxicity evaluations were performed to assess the biocompatibility and safety of the produced RGO. Cell viability assays were conducted using DLD-1 and CHO cell lines to determine the potential cytotoxic effects of RGO produced by each bacterial strain. Additionally, apoptotic, and necrotic responses were examined to understand the cellular mechanisms affected by RGO exposure. The results indicated that all the RGOs have concentration-dependent cytotoxicity. A significant amount of cell viability of DLD-1 cells was observed for *L. lactis* reduced graphene oxide. However, the highest cell viability of CHO cells was observed for *L. plantarum* reduced graphene oxide. All reduced graphene oxides have low apoptotic and necrotic responses in both cell lines. These findings highlight the importance of considering the specific bacterial strain used in RGO production as it can influence the toxicity and cellular response of the resulting RGO. The toxicity and cellular response to the final RGO can be affected by the particular bacterial strain that is employed to produce it. This information will help to ensure that RGO is used safely in a variety of applications, including tissue engineering, drug delivery systems, and biosensors, where comprehension of its toxicity profile is essential.

## Introduction

Graphene is a hexagonally structured carbon material with numerous properties that are applicable in different areas.^1−3^ Graphene oxide (GO) and reduced graphene oxide (RGO) are oxygen-carrying graphene derivatives that have attracted the attention of researchers due to their intense biological applications.

Graphene production usually begins with graphite to obtain graphite oxide. Graphite oxide has a layered structure similar to graphite, but the planar carbon atoms in it are decorated by different oxygen-containing groups that not only expand the interlayer distance but also make the layers hydrophilic. If these layers are separated by ultrasonication, the structure with one or more layers is called graphene oxide (GO). Reduced graphene oxide (RGO) is the form of GO whose oxygen content is reduced by chemical, thermal, and other methods.^4^ The most important goal of all reduction processes is to produce graphene-derived materials that are similar in structure and properties to the pristine graphene structure. A major challenge associated with the reduction process is the significant alteration that occurs in the structure of the carbon plane due to even residual functional groups and defects, making appropriate reduction procedures that will approximate the pristine graphene structure and properties necessary. Graphene is generally obtained from GO by reduction, as well as by micromechanical exfoliation of pyrolytic graphite,^1^ epitaxial growth,^5,6^ and chemical vapor deposition methods (CVD).^6,7^ For comparison purposes, GO and RGO are known to have two important properties: (1) they can be produced with cost-effective chemical methods using cheap graphite as the raw material and (2) they can form highly hydrophilic and stable aqueous colloids. These two important properties allow simple and inexpensive solution processes for large-scale use of graphene and facilitate the assembly of macroscopic structures. Therefore, the reduction of GO is a key issue affecting the performance of products that consist of RGO. Although it is difficult to reach perfect graphene, the results of the research bring it closer to us.

The reduction of GO to produce RGO can be achieved by a thermal,^8^ electrochemical,^9^ or chemical^10^ method. The chemical reduction method is seen as a promising approach for the production of large quantities of graphene since it is a simpler process compared to other methods, requires less equipment, and is economical.^10^ In this method, graphite is oxidized to graphene oxide (GO), and then the GO is reduced to graphene using strong reducing chemical agents. It has been reported that various reducing agents, such as hydrazine^11^ and sodium hydride, can be used to reduce GO. However, these materials are potentially toxic, highly dangerous, and may leave behind chemical residues or contaminants on RGO that can have adverse effects on human health and environment.^12^ Even minute quantities of hazardous substances can be harmful, especially when used in biological applications. GO contains various oxygen-containing functional groups such as carboxyl, hydroxyl, and epoxy groups. Chemical reduction methods may not effectively remove all these functional groups, resulting in residual oxygen functionalities that can affect the electrical, thermal, and mechanical properties of the RGO, limiting its performance in certain applications. On an industrial scale, the handling of the hazardous waste produced by the reduction reaction may dramatically raise the cost. Poor processability is another barrier to the use of chemically reduced graphene oxide in practical applications because, without a modification step (both covalent and noncovalent), RGO tends to aggregate irreversibly due to strong van der Waals forces between the graphene planes.^13^ In thermal reduction, the quality of the RGO is dependent on the thermal treatment conditions, which call for high temperatures up to 2000 °C under certain environments, such as argon and hydrogen, making it critical and occasionally crucial to the product quality. Thermal reduction methods are used as an efficient alternative route, typically to reduce the GO directly to produce the RGO. Furthermore, the GO is hydrophilic as well, making it difficult to separate it from the aqueous medium because any treatment other than freeze-drying results in an uncontrolled partial reduction, which will affect the final product’s quality and the required level of production repeatability when using thermal reduction.^14^ Even though thermal annealing appears as an effective method in product quality, there are several drawbacks of the method. Like chemical reduction, thermal reduction may result in limited control over the reduction process. The temperature and duration of the treatment can influence the degree of reduction and final properties of the RGO. There is a possibility of structural damage due to high temperatures used can cause induce structural damage to the graphene lattice. Excessive heat can lead to the formation of defects, such as vacancies, wrinkles, or structural rearrangements, which can affect the electrical, mechanical, and chemical properties of RGO. Restacking due to van der Waals forces results in reduced surface area, decreased electrical conductivity, and limited accessibility of the RGO material, which can impact its performance in certain applications. The thermal reduction process typically requires high temperatures, which can consume a significant amount of energy. Scaling up the process for large-scale production may have challenges in terms of energy efficiency and cost-effectiveness. There are also safety concerns associated with high temperatures employed.

Electrochemical reduction appears as one of the green reduction methods,^15^ but it has its own drawbacks to be considered. During the electrochemical reduction, the electrode used can become fouled or passivated due to the deposition of reaction byproducts or impurities. This fouling can affect the efficiency and effectiveness of the reduction process. The choice of the electrolyte used is crucial. Some electrolytes may contain impurities or ions that can contaminate the resulting RGO. The process is needed for precise control of various parameters, including applied potential, current density, and electrolyte composition. Small deviations in optimal control parameters can lead to variations in the degree of reduction and quality of the RGO produced. Scaling up the electrochemical reduction process while maintaining uniform reduction throughout a large area can be complex. Prolonged electrochemical reduction can cause electrode degradation or wear, potentially affecting the performance and reliability of the reduction process. The use of high voltages and currents also brings in safety considerations.

Recently, attempts have been undertaken to use natural products instead of harmful reducing chemicals to address the aforementioned issues. To remove all these drawbacks, the green reduction method has been developed by using environmentally friendly, nontoxic reductants. Green reducers used in this method include green tea,^16^ glucose,^17^ vitamin C,^18^ parsnip root,^19^ and bacteria.^20^

Green chemical reduction methods may exhibit lower reduction efficiencies compared to more aggressive chemical or thermal reduction processes. The use of milder reducing agents can result in incomplete reduction or variations in the degree of reduction, affecting the quality and properties of the resulting RGO. Achieving precise control over the reduction process can be challenging, leading to difficulties in replicating results and obtaining consistent product characteristics. For instance, ascorbic acid (vitamin C) works well in reducing GO, but without an additional stabilizer, the result typically displayed a highly agglomerated shape.^21^ Although the resulting RGO produced a stable aqueous solution, reducing sugar or dextrose and protein bovine serum albumin^22^ have also been used in the reduction of GO. However, due to their poor reducing capabilities, an alkali is required as a coreductant. While green reducing agents are generally considered more environmentally friendly, they may still introduce impurities or byproducts during the reduction process. Depending on the specific reducing agent used, these impurities may affect the purity, stability, and performance of the RGO, especially in applications that require high levels of purity or specific material properties. Green chemical reduction methods may face challenges when scaling up to industrial or large-scale production. The availability, cost, and extraction of green reducing agents in large quantities can be limiting factors. Additionally, the use of specific reaction conditions or additional purification steps can add complexity and increase production costs. Therefore, it is still extremely desirable to have a reliable, affordable, and environmentally friendly reducing agent for the chemical production of soluble graphene in large quantities.^21,23,24^

The manufacture of RGO using microbial techniques has become a more secure option in recent years. These procedures make use of microorganisms like bacteria or fungus that function naturally as bioreduction agents. The microorganisms act on GO, successfully converting it to RGO through enzymatic or metabolic activities. Using microbial methods has several benefits over chemical and thermal treatments, notably in terms of safety. The inherent safety of the microbial technique is one of its main benefits. The health of humans is rarely endangered by microorganisms, which are typically nontoxic. The use of microorganisms as bioreduction agents eliminates the need for dangerous chemicals, making the process much safer for both employees and the environment. The energy needs and risk of accidents related to high temperatures and toxic solvents are also decreased by using microbial techniques, which are normally used in mild reaction conditions. There are many ways to produce RGO, including chemical/thermal and microbiological processes. The microbial technique offers a safer alternative to chemical methods, which have been widely employed because of how easily they can be applied. The microbial technique reduces safety hazards and environmental problems by using microorganisms’ enzymatic or metabolic capacities rather than risky substances like glucose and ascorbic acid.

Compared to chemical approaches that utilize potentially dangerous materials, microbial reduction techniques are fundamentally safer. Even though chemical reduction methods employed less hazardous substances such as glucose or dextrose or ascorbic acid for reducing GO, these substances can still have associated risks, including toxicity, flammability, or release of toxic gases. After reduction, they may leave behind some residual functional groups. The high temperatures or chemical agents used in these methods can introduce defects, such as structural disorder, vacancies, and lattice distortions in the resulting RGO. Microorganisms, including bacteria and fungus, are harmless, naturally occurring substances that rarely endanger human health. The use of microorganisms as bioreduction agents eliminates the need for potentially dangerous chemicals like glucose, resulting in a safer production process for both the environment and employees. The use of sustainable, microbial reduction techniques is good for the environment. Unlike often used chemical procedures, they do not call for the use of dangerous solvents or produce hazardous waste. Under mild reaction conditions, microorganisms may effectively convert graphene oxide (GO) to reduced graphene oxide (RGO), lowering energy requirements, and limiting the environmental impact associated with high-temperature operations. Mild reaction conditions, like a neutral pH and room temperature, are usually favorable for microbial reduction. In addition to ensuring the process’s safety, this tactful method also protects the RGO’s structural integrity and desired qualities. The reduction process facilitated by microorganisms can preserve the original graphene sheet structure to a large extent, leading to RGO sheets that maintain a relatively high level of graphitic ordering. Chemical/thermal reduction methods can cause more significant structural changes in the graphene oxide (GO) lattice. The production of high-quality RGO with improved characteristics suited for a variety of applications can be achieved by microbial reduction by avoiding severe conditions. Microbial reduction tends to result in RGO with a higher degree of oxygen functional group removal. The enzymatic action of microorganisms can selectively target and reduce the oxygen-containing functional groups on GO, leading to a more complete removal of these groups in the resulting RGO. Chemical/thermal reduction methods can remove a significant portion of the oxygen functional groups present in GO, but they may leave behind some residual functional groups. The selectivity of chemical agents used in these methods may not be as high as the enzymes produced by microorganisms, resulting in a partial removal of oxygen functional groups. RGO obtained through microbial reduction methods generally exhibits lower electrical conductivity compared to chemically/thermally reduced RGO. The presence of residual oxygen functional groups in microbial RGO can limit electron mobility and impede the formation of a highly conductive network. Chemical/thermal reduction methods tend to yield RGO with higher electrical conductivity. The removal of a larger portion of oxygen functional groups can enhance the conductivity of the RGO by facilitating the formation of a more interconnected graphitic network.

Scalability and cost-effectiveness of microbial reduction techniques have been proven. To satisfy manufacturing objectives, microorganisms may be scaled up and simply grown. Economically, microbial approaches for RGO production are feasible due to the accessibility of low-cost growth media and the possibility of bioreduction in large-scale bioreactors. Biocompatible RGO is essential for applications in biomedicine and biosensing, and it can be produced using microbial reduction techniques. It is ensured that the produced RGO is devoid of hazardous residues that can impair its biocompatibility by using microorganisms as bioreduction agents. In drug delivery systems, tissue engineering, and biomedical imaging, this creates prospects for RGO-based materials. In terms of the variety of microorganisms available and the potential for genetic engineering, microbial reduction techniques offer flexibility. Unique enzymatic activities of various microbes enable customized reduction processes and the generation of RGO with particular features. The spectrum of RGO properties that can be achieved can be further increased by using genetic engineering approaches to increase the enzymatic activity of microorganisms. In conclusion, the microbial reduction of GO has a lot of benefits, such as increased safety, environmental friendliness, moderate reaction conditions, scalability, cost-effectiveness, biocompatibility, and adaptability. The manufacture of high-quality RGO with a variety of applications in numerous fields is made attractive and promising by these benefits, which make microbiological techniques.^25,26^

Recent reports on microbial reduction have shown that GO is the terminal electron acceptor for bacterial organisms, and reduction of GO has been shown to be possible by microbial effects during respiration or the electron transfer process between bacteria and GO. In the microbial reduction process, bacteria can take organic and inorganic molecules from the environment and convert them into substances necessary to initiate the cellular process. In this process, the oxidation–reduction mechanism is used to obtain an energy source. There exist studies in the literature on RGO production by *Shewanella*,^27^*Bacillus subtilis*,^28^*Escherichia coli*,^29^*Gluconacetobacter xylinus*,^25^*Shewanella oneidensis*,^30^*Lactobacillus plantarum*^31^ and *Lactococcus lactis*.^32^ These studies showed that the reduction reaction mechanisms between GO and bacteria depend on the bacterial cell structure. It is noted that the bacterial cell structure influences the ability to hydrolyze acidic groups directly or indirectly, particularly groups containing oxygen atoms and attached to the GO molecular structure. It has been claimed that, depending on the kind of bacteria used, it is possible to select a procedure for effectively degrading GO based on the desired requirements of the final nanomaterial.^28^ The amphiphilic character of the GO and RGO layers makes them valuable for their use in biomedical applications, especially their interactions with cells.

RGO has recently been studied for biological applications such as drug carriers, diagnostic sensors, biomarkers, and antibacterial agents.^33^ However, it has been reported that it may cause many adverse effects in vitro, including the generation of reactive oxygen species (ROS), cell apoptosis, inflammatory cytokines, loss of membrane integrity, membrane distress caused by direct contact with sharp edges of RGO, and inflammatory cell infiltration.^34^ Recent studies have also demonstrated that RGO is possibly toxic and can reduce integration into cell membranes, inducing apoptosis in a dose-dependent manner.^35,36^ It is therefore essential to understand the toxicological mechanisms required to improve the bioavailability of RGO as well as to investigate the effects on the safety of living systems. This will enable us to find safer and minimal or nontoxic methods for preparing RGO for biomedical applications.

Based on the above analysis, this study focused on the microbial production of reduced graphene oxide using graphite-derived graphene oxide as a starting material. The method provides a safe, clean, and efficient means of reducing graphene oxide and information about their interaction with cells, which is needed in various bioengineering applications. Three different microorganisms were used for the reduction process to prepare RGO from chemically derived GO, with each RGO characterized to understand how their physical and chemical properties change. The toxic effects of these materials on DLD-1 and CHO cell lines and their apoptotic/necrotic responses were also investigated.

## Materials Methods

### Bacterial Strains and Culturing

*Escherichia coli* ATCC 10536, *Lactococcus lactis* DSM 20481, and *Lactobacillus plantarum* CCM 1904 were supplied from the American Type Culture Collection, the German Collection of Microorganisms and Cell Cultures GmbH (DSMZ), and the Czech Collection of Microorganisms, respectively. *E. coli* and *L. lactis* were cultured in Nutrient Broth (NB), and *L. plantarum* was cultured in CCM Medium No 6.

The following path was followed in the preparation of precultures: 5 mL of medium was added to a 25 mL bottle, after which microorganisms (*E. coli*, *L. lactis*, and *L. plantarum*) were inoculated by a single colony and kept on a rotary shaker for 18 h. Incubation temperatures were 37 °C for *E. coli* and 30 °C for *L. lactis* and *L. plantarum*. The second preculture was prepared in 500 mL flask that has 150 mL of medium inoculated with the first preculture at 1% (v/v). Incubation was carried out on a rotary shaker at 30 or 37 °C, depending on the microorganism, for 24 h.^31,32^ The cells were harvested by centrifugation and washed twice with phosphate buffered saline (pH 7.2). Thereafter, 200 mg of wet cells were collected in a 50 mL tube to be used later in the reduction process.

### Preparation of Graphene Oxide

Graphite powder, potassium permanganate (KMnO_4_), nitric acid (HNO_3_), sulfuric acid (H_2_SO_4_) (98%), hydrogen peroxide (H_2_O_2_) (30%), hydrochloric acid (HCl) (36%), and ethanol were all purchased from Sigma-Aldrich (USA) and used directly without further purification.

GO was prepared using the modified Hummer method. According to this method,^37^ 2 g of graphite powder was added to a solution of 80 mL of H_2_SO_4_ and 20 mL HNO_3_ and stirred in an ice bath. Then, 12 g of KMnO_4_ was slowly added to this mixture, 2 g at a time, and the temperature was raised to 35 °C under vigorous stirring. After half an hour of stirring, 160 mL of deionized water was added to the mixture for dilution, and then it was left to rest for 1 h. It was then diluted with another 400 mL of deionized water, followed by the slow addition of 12.0 mL of H_2_O_2_. After all these processes, it was observed that the black graphite suspension turned into a bright yellow graphite oxide solution. The solution was centrifuged at 3000 rpm/min for 15 min to obtain a graphite oxide precipitate and washed with deionized water. After the last wash, the pellet was resuspended in water to obtain an aqueous graphite oxide solution. A graphene oxide solution (6 mg/mL) with a single or multiple layers was obtained by the exfoliation of stacked layers in a graphite solution by sonification for 2 h. This solution was used in the rest of the experiments.

### Microbial Reduction of Graphene Oxide

The solution of GO with 0.5 mg/mL concentration was prepared and 200 mg bacterial biomass was added. The prepared solutions were incubated at 37 °C for *E. coli* and 30 °C for *L. plantarum*(31) and *L. lactis*(32) for a week. Aerobic reductions were carried out until stable black dispersions were observed. When the reaction was complete, cells were disrupted with a 5 min sonification, and the black precipitate was separated with centrifugation at 10,000 rpm for 10 min and resuspended in water. Purification was continued with sequential washings of 80% ethanol and 1 N HCl. Water washings were applied between steps, and the last washing continued until neutralization. Neutralized samples were lyophilized and used for characterization. The samples were named ERGO for *E. coli* reduced graphene oxide, LLRGO for *L. lactis* reduced graphene oxide, and LPRGO for *L. plantarum* reduced graphene oxide.

### Characterizations of Graphene Oxide and Reduced Graphene Oxides

Physicochemical characterizations of GO, ERGO, LLRGO, and LPRGO samples were performed by Fourier transform infrared spectroscopy (FTIR), microconfocal Raman spectroscopy, X-ray diffraction (XRD), X-ray photoelectron spectroscopy (XPS), and thermogravimetric analysis (TGA)

FTIR analysis was carried out on a Shimadzu IR Prestige 21 for the detection of chemical structures of samples and surface functionalities. Samples were prepared in potassium bromide (KBr) pellets, and spectra were recorded between 400 and 4000 cm^–1^. Microconfocal Raman spectroscopy was employed for further chemical analysis and defect characteristics of samples. The spectra were recorded from 200 to 3000 cm^–1^ on a Renishaw Invia Raman Microprobe by using a 532 nm argon ion laser. XRD, Rigaku MinFlex, D/max 2550-PC with Cu-Ka radiation (λ = 0.15406 nm) was employed for the determination of oxidation and reduction of samples and to detect defects. Data were collected between scattering angles (2θ) of 0–90° at a scanning rate of 2° min^–1^. The TA Instrument Thermogravimetric Analyzer Q50 (USA) was used for determining the decomposition of samples under a N_2_ atmosphere. For analysis, samples were heated from room temperature to 600 °C at a 5 °C/min heating rate, and mass losses were recorded as a function of temperature. X-ray photoelectron spectroscopy (XPS) measurements were carried out on a SPECS XP Flexmod (Germany) equipped with a PHOIBOS hemispherical energy analyzer and monochromatic Al Kα X-ray excitation (*h*ν = 15 kV, 400 W). Binding energy (BE) calibration of the XP spectra was carried out with the help of the amorphous carbon C1 signal located at 284.3 eV. C 1s and O 1s spectra of samples were recorded with the pass energy *E*_p_= 45 eV.

### Influence of GO and RGO on Cell Viability

#### Materials

CHO and DLD-1 cell lines were purchased from ATCC. Dulbecco Modified Medium (DMEM; Biological Industries), Fetal Bovin Serum (FBS; Biological Industries), l-glutamine, and Penicillin/Streptomycin were used as complete cell culture mediums. Cells were removed from the culture dishes and washed with trypsin-EDTA (trypsin-ethylenediamine tetraacetic acid) and phosphate buffer (PBS). Cells were counted using trypan blue. In a cytotoxicity test, cell viability was determined by using MTT, a tetrazolium salt. In dual staining, apoptosis/necrosis ratios were determined using Ribonuclease-A, Propidium Iodide (Serva, Israel), and Hoechst 33342 (Serva, Israel). All cell culture studies were performed in culture dishes and multiwell plates (Corning, USA).

#### Cell Culture

The frozen cells were thawed at 37 °C in a short time. Cells dissolved in a sterile laminar flow cabinet were transferred to a 15-mL falcon tube and centrifuged at 2500 rpm for 2 min. Three milliliters of DMEM medium (containing 10% FBS and 1% antibiotic) was placed in the flask, and 25 cm^2^ flasks were cultured after homogenization. The flask was incubated with 5% CO_2_ at 37 °C.

#### Cytotoxicity Test (MTT)

Ninety-six-well plates were used for the toxicity test. After counting the number of living cells, calculations were made to place 5 × 10^3^ cells in each well. In 96-well plates, 100 μL cells were placed in each well and left for incubation for 24 h. After 24 h, cells were checked for adherence to the well plate surface. The medium in the wells was emptied. RGO samples were prepared at 1 mg/mL. Three wells were added to the well plates from the top to the bottom, at concentrations of 200, 100, 50, 25, and 12.5 μg/mL. Only the medium was placed in the negative control group. DMSO (5%) was placed in the positive control group and incubated for 24 h. At the end of the incubation, the medium in the well plates was removed. MTT (1 mg/mL) solution was added to 50 μL wells. After incubating at 37 °C for 2–2.5 h, the MTT solution in the wells was drained and 100 μL of the MTT solvent (isopropanol) was added. The absorbance density values of 96-well plates were determined at 570 nm in an ELISA plate reader for the detection of cell viability.

#### Double Staining

Cells were cultured in 48-well plates with 10 × 10^3^ cells per well. Incubation was for 24 h. At the end of 24 h, the medium in the wells was emptied and the samples were studied for different concentrations (100 and 25 μg/mL) in three replicates. Only the medium was added to the negative control group cells and added to the positive control group with 5% DMSO in the medium. Incubation was 24 h. After incubation, the medium in the wells was emptied, and 70 μL of double-staining working solution was added to each well. Forty-eight-well plates were closed so that they could not see any light and were incubated for 15 min. At the end of the incubation, apoptotic cells and FITC (480–520 nm wavelength) necrotic cells were evaluated by using a DAPI filter in a fluorescence microscope (fluorescence inverted microscope, Leica DM6000B, Sweden). The dual staining method stains the nucleus, thus showing apoptosis and necrosis. Ribonuclease A is used and stored at −20 °C (Sigma R-500). Ribonuclease A does not stain RNA. In this way, it destroys the cytoplasmic RNA. Hoechst 33342 staining dye solution is stored at +4 °C. It stains apoptotic cells. In this way, true apoptotic cells are determined. Propidium iodide is stored at +4 °C. It stains both DNA and RNA. When stained with red, it shows secondary necrosis. When stained with propidium iodide fluorescent dye in the double staining solution, the nucleus cells are seen in red under red fluorescent and green fluorescent light, indicating that the cells have undergone necrosis. The number of apoptotic and necrotic cells was counted from 10 randomly selected microscopic fields, and the results were calculated as a percentage by proportioning the number of normal, apoptotic, and necrotic cells to the total number of apoptotic and necrotic cells. This experiment was repeated three times.

## Results and Discussion

In this study, RGOs were prepared in two steps. First, chemical synthesis of GO from graphite dust by modified Hummer’s methods;^37^ second, microbial reduction of GO by three different microorganisms: *E. coli*, *L. plantarum*, and *L. lactis*. Since the reduction mechanisms of the microorganisms were different from each other, they have been effective on different functional groups on the GO basal plane and at the edges, thus affecting the physicochemical behavior of the material. It was also intended to determine how these different RGOs influence cell viability and whether they cause any apoptosis or necrosis.

### Characterizations of RGOs

Although the same GO was used as the starting material in production of RGOs, the final products had different characteristics. The physicochemical properties of products were examined by Raman, FTIR, XRD, XPS, and TGA.

Raman spectroscopy is the most reliable technique for the structural characterization of graphitic materials.^38^ In this method, the analysis of the three main peaks of the GO and RGO spectra, as well as the peak positions, peak widths, and relative intensity ratios (*I*_D_/*I*_G_), is important.^39^ D, G, and 2D bands are the main bands and were located at 1350, 1580, and 2680 cm^–1^, respectively.^40^ The G band appeared as a result of in-plane vibration of the sp^2^ carbon atoms, while the D band is related to the crystal distortion caused by sp^3^ defects, and the 2D band shows an overtone of the D band caused by the disturbance. For single-layer graphene, the 2D band appears as a sharp and symmetrical peak, but with increasing thickness, it expands more like a few layers of graphene.^41^

**Figure 1:**
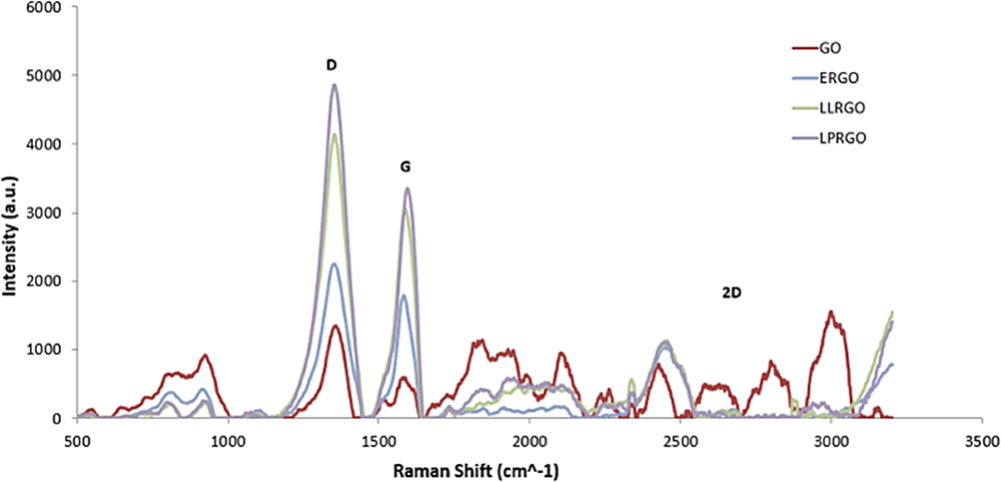
Raman spectrum of GO and RGOs obtained by different microorganisms *E. coli* (ERGO), *L. lactis* (LLRGO) Reproduced from ref (32). Copyright 2020 Celal Bayar University Journal of Science, *L. plantarum* (LPRGO) Reproduced from ref (31). Copyright 2019 International Journal of Nanoscience and Nanotechnology.

Figure 1 shows the Raman spectra of GO, ERGO, LLRGO, and LPRGO. The D peak at around 1350 cm^–1^ indicated the presence of defects in all the samples. The appearance of a large and dense 2D band indicated that GO has more defects compared to ERGO, LLRGO, and LPRGO samples. The G band for GO was at 1590 cm^–1^ and for ERGO, LLRGO, and LPRGO, it was at 1587, 1583, and 1597 cm^–1^, respectively. A wavenumber and density increase in the G peak was observed because of an increase in the number of layers. As shown in Figure 1, it is seen that GO has a fairly low density compared to other samples in which the number of layers increased. It can be speculated that the layers separated by reduction resat on top of each other. When the ratio between D and G peak intensities was compared, *I*_D_/*I*_G_ was found to be 2.41 for GO and 1.26, 1.35, and 1.46 for ERGO, LLRGO, and LPRGO, respectively. These values were found to be in good agreement with the literature.^29,31,32^ Results obtained from Raman clearly showed that all three microorganisms effectively reduced GO; however, the layering capacity of each microorganism was different and produced RGO with different properties. It is estimated that depending on the reduction mechanism and enzymes used in their respiratory systems, each microorganism differently reduced some of the oxygen containing functional groups. *E. coli* has reductase bd- and bo- type cytochromes, such that both can reduce oxygen.^42^*L. lactis* generates protons by a heme-dependent aerobic electron transport chain, and heme induces respiration in *L. lactis*. They use NADH as an electron donor and oxygen as an electron acceptor. Heme is an essential cofactor of cytochrome complexes in the electron transport of respiring cells.^43^*L. plantarum* is capable of using oxygen or nitrate as a terminal acceptor. It has oxygen and nitrate-dependent respiration, and they have bd-type cytochrome and the nitrate reductase.^44^ This difference in reduction of functional groups was affected by the distance between layers, depending on the reduced molecules. In addition, it is thought that the separated layers of ERGO, LLRO, and LPRGO, which increased in the G peak wave numbers as a result of the reduction process, were piled up on each other again.

**Figure 2:**
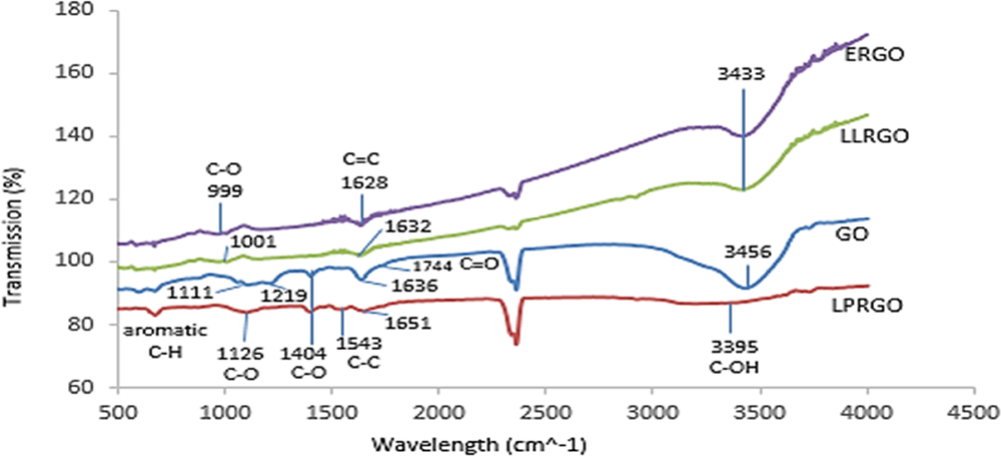
FTIR spectrum of GO and reduced graphene oxides; ERGO, LLRGO, and LPRGO.

FTIR spectroscopy was used for determining different types of functional groups formed in GO and RGO.^45^ The FTIR spectrum of GO (Figure 2) showed a broad peak at 3000–3600 cm^–1^ originating from stretching vibrations of the −OH group, and a very small shoulder observed around 1744 cm^–1^ belongs to the stretching vibrations of carboxyl peaks (C=O). Aromatic C=C peaks appeared at 1636 and at 1396 cm^–1^, and C–O carboxyl and 1219–1111 cm^–1^ stretching vibrations of epoxy C–O–C and alkoxy C–O groups were determined. Reductions, as seen in Figure 2, accomplished by *E. coli*, *L. Lactis*, and *L. plantarum* were observed as the removal or intensity reduction of some absorbance peaks. Peaks around 3000–3600, 1744, 1396, and 1219–1111 cm^–1^ have decreased and disappeared dramatically, and a single C–O vibrational stretching band at 999 and 1001 cm^–1^ formed from remaining carboxyl or alkoxy groups for ERGO and LLRGO. This result showed that both *E. coli* and *L. lactis* similarly reduced GO by attacking the same functional groups in their respiratory systems. *E. coli* and *L. lactis* mostly reduce epoxy as well as some alkoxy and carboxyl groups. For LPRGO, the −OH peak at 3400 cm^–1^ almost disappeared, alongside the 1620 cm^–1^ peak of the 1720 cm^–1^ C=O carboxyl shoulder and the 1111 cm^–1^ C–O alkoxy, while the 1404 cm^–1^ C–O carboxyl peak remained the same as in GO; however, the 1219 cm^–1^ C–O–C epoxy peak was completely lost. It was concluded that *L. plantarum* most effectively reduced alkoxy groups, as seen in Figure 2. These results have shown that all three microorganisms were effective in reducing the functional groups on GO and that different microorganisms used different oxygen groups of GO in their respiratory systems. These findings agree with the *I*_D_/*I*_G_ results obtained from Raman.

**Figure 3:**
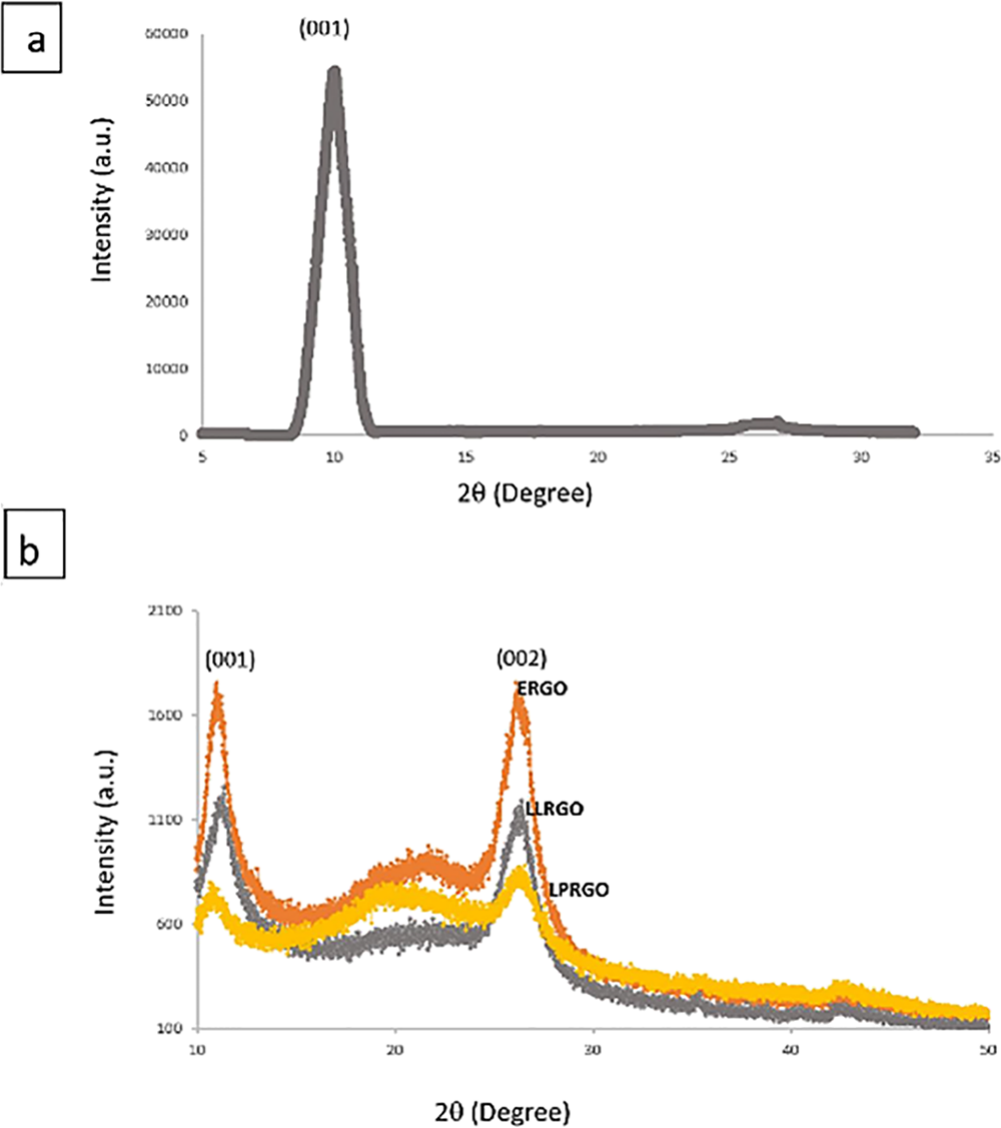
XRD spectrum of (a) GO, (b) ERGO, LLRGO, and LPRGO.

XRD patterns of GO, ERGO, LLRGO, and LPRGO nanosheets are presented in Figure 3. The characteristic peak (002) of graphite around 26° almost disappeared after oxidation, while the observed peak at 9.98° corresponds to the (001) diffraction peak of GO. The *d*-spacing of GO was calculated from Bragg’s equation as 0.89 Å.^46^ The large interlayer spacing of GO came from the formation of oxygen functional groups such as hydroxyl, epoxy, and carboxyl. As a result, almost all the graphite was oxidized. After microbial reduction of GO, some oxygen functional groups were removed and gave 2θ at around 26°, while some functional groups stayed on graphene and kept 2θ at around 11°. Interlayer spacing of ERGO 0.340, LLRGO 0.336, and LPRGO 0.342 were found. A decrease in interlayer spacing indicates a successful removal of oxygen functional groups. When the XRD spectra of ERGO, LLRGO, and LPRGO were examined, it was seen that there was quite a high peak in the range of 15–24°, which came from the short-order range of stacked layers for LPRGO; however, for ERGO and LLRGO, the peak either decreased or disappeared, indicating that stacked layers were not observed for RGOs formed by *E. coli* and *L. lactis*. It can be said that the XRD results agreed with the FTIR and Raman results obtained.

**Figure 4:**
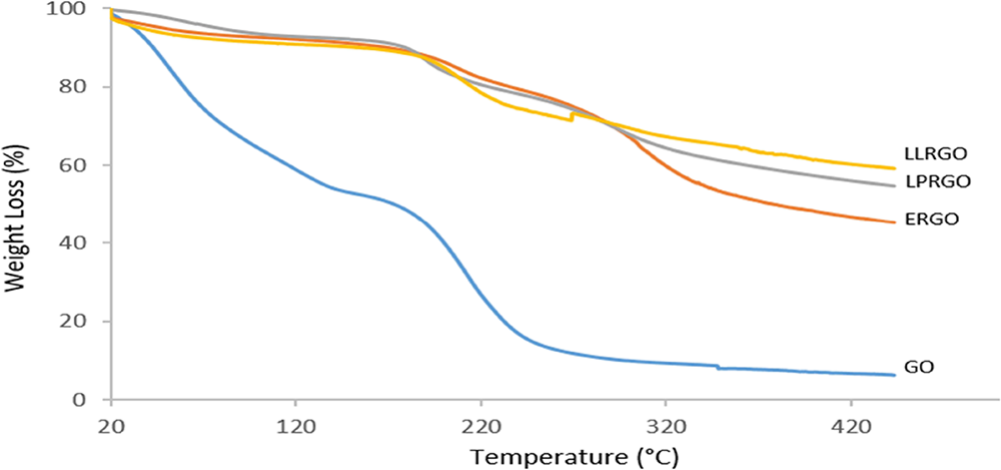
TGA analysis of GO, ERGO, LLRGO and LPRGO.

The thermal stability of GO, ERGO, LLRGO, and LPRGO was examined by TG analysis, as shown in Figure 4. GO decomposition occurred mainly in three steps, implying a high degree of oxidation. The first weight loss of GO at 20–122 °C was around 42% and belonged to the evaporation of adsorbed water molecules in between graphene layers. At this region, all the reduced graphene oxides (ERGO, LLRGO, and LPRGO) exhibited similar degradation, with weight losses of around 18% as well as water losses. The second step observed at 122–307 °C with a weight loss of around 50% was due to the loss of oxygen-containing functional groups, and the third step above 307 °C with a weight loss of 4% was related to unstable carbon remaining in the structure and the pyrolysis of oxygen functional groups in the main structure to yield carbon dioxide, carbon monoxide, and water.^41,47−49^ At the second step, ERGO and LPRGO showed high and steep decomposition with weight loss of around 28 and 16%, respectively. However, a weight loss of 18% was observed for LLRGO. At the third region, ERGO with 9% weight loss had a steeper curve compared to LLRGO and LPRGO. Both LLRGO and LPRGO had 5% weight loss in this region. All RGOs, i.e., ERGO, LLRGO, and LPRGO, exhibited similar characteristics but had lower weight losses compared to GO, which can be attributed to the smaller amount of oxygen functional groups in their structures. Comparison of the reduction efficiency of the three microorganisms using TG analysis shows that ERGO had a higher decomposition rate with a total weight loss of 54%, while LLRGO and LPRGO had weight losses of 41 and 45%, respectively. It could be concluded that *L. lactis* is very effective for reducing GO by removing more oxygen functional groups compared to *L. plantarum* and *E. coli*.

**Figure 5:**
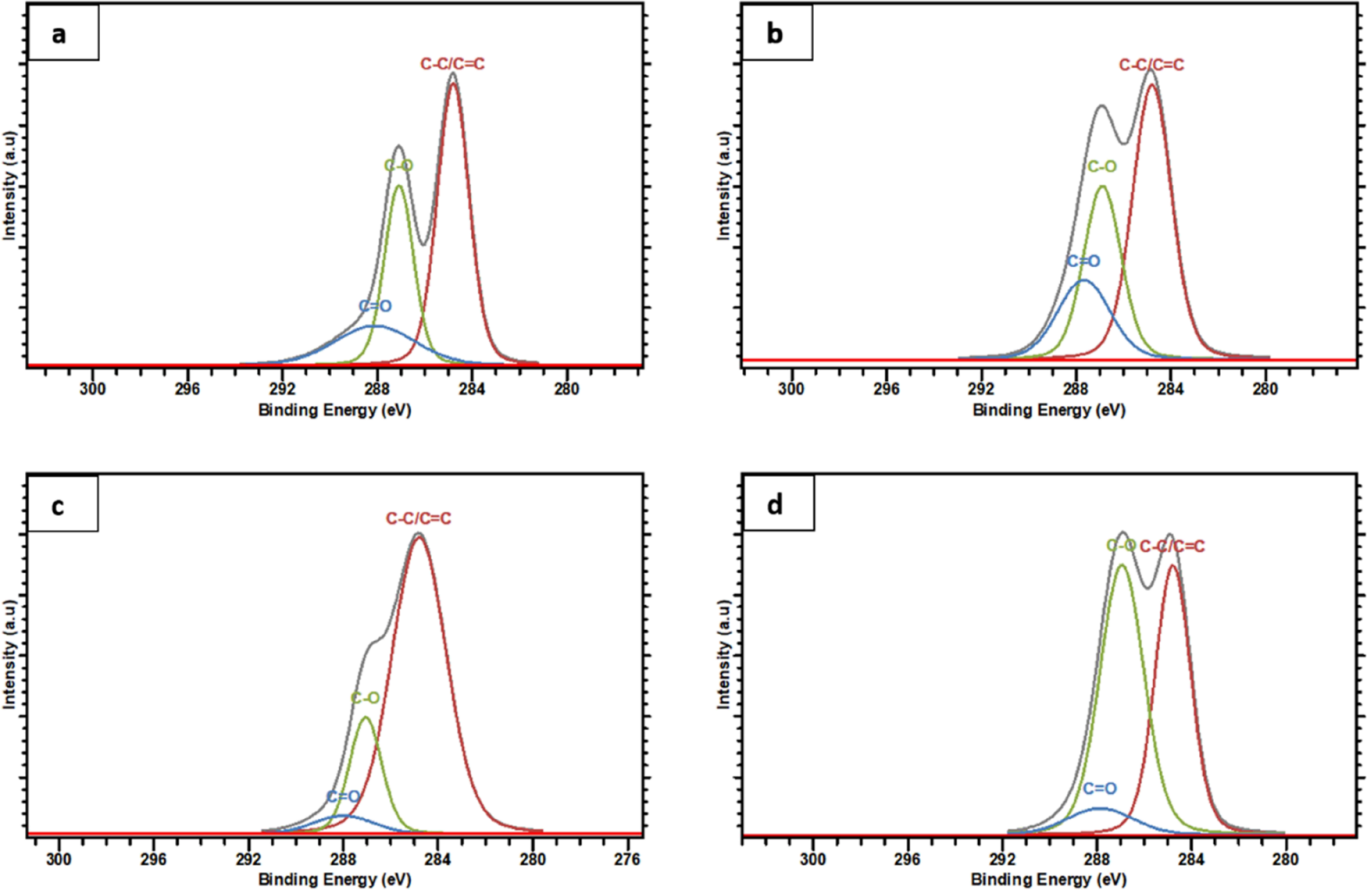
XPS analysis of (a) GO, (b) ERGO, (c) LLRGO, and (d) LPRGO.

XPS analysis was carried out for GO and all RGOs to analyze the impact on the carbon content of different bacteria reduction processes. Figure 5 shows the C 1s spectra taken from all the samples and the deconvoluted C 1s peaks after Gaussian–Lorentzian fitting by peak processing software. The GO sample presented a broad tail toward high energies because of oxygen groups in the structure, as shown in Table 1.This tail was constituted from different carbon bonding configurations: C–C (for sp^3^)/C=C (for sp^2^) ∼284.8 eV, C–O ∼287 eV, and C=O ∼288 eV, as seen in Figure 5. By reduction, the peak ratios of the different peaks changed; C–C/C=C contents increased, whereas the oxygen functional group content decreased, as indicated by the C 1s/O 1s atomic ratio, as seen in Table 1. As the reduction improved from GO to RGO, the C–C/C=C content increased in intensity. The findings were consistent with the reduction in oxygenated functional groups seen in the O 1s spectra for all samples. Results showed that *L. lactis* was more efficient in reduction compared to *L. plantarum* and *E. coli*; however, it did not improve graphitization as much as *E. coli*. XPS results were found to be consistent with TGA results by increasing the reduction efficacy of bacteria *L. lactis* > *L. plantarum* > *E. coli*.

**Table 1:** XPS Spectra of GO and all RGOs

samples in atom %	C–C/C=C	C–O	C=O	C 1s	O 1s	C 1s/O 1s atomic ratio
GO	35.17	20.5	12.50	68.24	31.76	2.15
ERGO	36.90	22.42	14.37	73.69	26.31	2.80
LLRGO	60.44	14.08	4.19	78.71	21.29	3.70
LPRGO	30.86	38.25	5.75	74.86	25.14	2.98

### Influence of GO and RGOs on Cell Viability

There are various physical and chemical properties of GO and RGO, such as solubility, dispersibility, layer number, lateral dimensions, sheet size, stiffness, defect density, and degrees of oxidation/reduction, all of which significantly influence their interactions with biological systems.^50^ These properties are determined by the reduction agent and influence the absorption, toxicity, and biodegradation of these products by cells.^51−53^ The synthesis method determines the size, morphology, solubility, toxicity, and biocompatibility of graphene. For biomedical applications, nanosized graphene materials must be synthesized to induce either toxicity or biocompatibility.

It was reported that the use of graphene and graphene-derived materials in biomedical research has been restricted because of latent cytotoxicity problems.^54^ Nowadays, researchers have started to measure the association of graphene and graphene-based materials with various cell lines and animal models to understand the mechanism of cytotoxicity.^26,55^ Gurunathan et al., have examined the biological properties of GO and RGO in different types of bacteria as well as cancerous and noncancerous cells.^56,57^ Chatterjee et al., studied the toxic effect of the oxygen level of GO and RGO on HepG2 cells.^58^ Jaworski et al., demonstrated that GO and RGO in different sizes have different toxicities to glioblastoma cell lines.^59^ Particle size and surface chemistry play a key role in the processes regulating cell interaction. Chemical changes on the surface of the GO and the formation of the corona protein have contributed to aqueous media dispersion and reduced agglomerate formation and size, enabling contact between materials and cells. Adhesion to the cell surface represents the first step in GO-cell membrane interaction. Electrostatic and steric associations with phospholipids, proteins, and saccharides have been confirmed to be important for GO and breakup adhesion of materials, both in model membranes and in the membranes of animal cells and bacteria.^18^

Chang et al. studied the cytotoxic effects of GO with different sizes on the A549 cell line, and they observed no signs of cytotoxicity.^60^ Hu et al. observed a loss of viability with a concentration of 200 μg/mL only with the smallest graphene sheets. The cytotoxicity of GO was low, in the range of 20–100 μg/mL concentration.^61^ It was observed that GOs have concentration-dependent toxicity toward different cell lines. Wang et al. showed that GO concentrations at 20 μg/mL or less have no toxicity, but 50 μg/mL concentrations or higher result in cytotoxicity caused by cell apoptosis.^62^ Lammel et al. demonstrated that toxicity in HepG2 cells occurs through different mechanisms, such as metabolic activity changes, plasma membrane integrity, and lysosomal function, dependent on GO and carboxyl-GO concentration and time.^63^ Pelin et al. observed cellular ROS production mediated mitochondrial depolarization induced GO and few-layer graphene (10 and 100 μg/mL) cytotoxicity.^64^

It has been reported that differences found in the literature are due to differences in physicochemical properties like surface functionalities and sizes of the graphene used in each work.^22,65^ Zhang et al. found that the cytotoxicity of GO in PC12 cells is concentration- and size-dependent.^65^ Zhang et al. reported that GO has concentration- and size-dependent cytotoxicity on PC12 cells.^65^ Akhavan et al. showed significant size-dependent cytotoxicity of RGO nanoparticles based on lateral size dimensions in human mesenchymal stem cells.^66^ Therefore, it is important to know the physical and chemical properties of graphene to understand its interaction with cells.^67^

In this study, the cytotoxicity of GO, ERGO, LLRGO, and LPRGO nanosheets was also investigated. Samples were dissolved in phosphate buffered saline (PBS) at a concentration of 1 mg/mL. Two different cell lines, Chinese Hamster Ovary Cells (CHO) and Colorectal Cancer Cell Line (DLD-1), were incubated for a period of 24 h with the GO, ERGO, LLRGO, and LPRGO dispersed in PBS at various concentrations (0, 12.5, 25, 50, 100, and 200 μg/mL). A MTT test was performed in order to quantify the toxicity. The results are tabulated in Tables 2 and 3 for DLD-1 and CHO cell lines, respectively.

**Table 2:** 24-h Cytotoxic Effects of Prepared Samples on DLD-1 Cells

	concentration (μg/mL)	%viability
Control		100
GO	200	75.4 ± 6.0
	100	100.0 ± 3.0
	50	103.04 ± 9.8
	25	107.94 ± 6.2
	12.5	110.7 ± 9.9
ERGO	200	78.8 ± 8.9
	
	100	87.0 ± 3.6
	50	90.7 ± 3.9
	25	99.4 ± 5.4
	12.5	102.3 ± 3.9
LLRGO	200	89.8 ± 3.5
	100	91.2 ± 5.0
	50	104.9 ± 2.2
	25	108,8 ± 4.3
	12.5	113.7 ± 2.9
LPRGO	200	87.4 ± 2.0
	100	89.3 ± 4.9
	50	98.1 ± 7.6
	25	101.4 ± 2.9
	12.5	102.2 ± 5.8

**Table 3:** 24-h Cytotoxic Effects of Prepared Samples on CHO Cells

	concentration (μg/mL)	%viability
Control	0	100
GO	200	69.4 ± 0.2
	100	76.4 ± 4.5
	50	84.9 ± 2.1
	25	90.4 ± 3.7
	12.5	96.6 ± 5.1
ERGO	200	69.9 ± 4.0
	100	75.1 ± 6.0
	50	74.4 ± 4.4
	25	81.0 ± 5.2
	12.5	81.9 ± 3.8
LLRGO	200	66.2 ± 1.1
	100	74.0 ± 1.9
	50	75.8 ± 4.7
	25	82.3 ± 4.9
	12.5	86.0 ± 3.5
LPRGO	200	69.9 ± 5.8
	100	72.6 ± 4.2
	50	89.4 ± 3.9
	25	94.8 ± 1.9
	12.5	104.1 ± 2.2

As seen in Table 2, most of the samples, including RGO, show little cytotoxic effect on DLD-1 cells. High cell viability (around 100%) was observed for GO up to 200 μg/mL. Reduction caused by microorganisms removed certain functional groups, and depending on this reduction, the physicochemical differences of ERGO, LLRGO, and LPRGO were observed. In this study, the effect of the removal of functional groups existing on RGO samples on the cytotoxicity of the DLD-1 cell line was not changed much depending on the microorganism employed. When cell viability was compared for the microorganisms, the highest cell viability was observed for LLRGO, and LPRGO and the values for these samples were the same as the values obtained for GO. ERGO samples had little toxicity to DLD-1 cells; however, viability was almost the same with LLRGO and LPRGO for concentrations below 25 μg/mL. In general, it could be implied that RGO samples were not toxic to the DLD-1 cell line at concentrations below 100 μg/mL.

The cytotoxic effects of GO and RGOs (ERGO, LLRGO, and LPRGO) on CHO cells were also examined, and in general, all the samples were found to be more cytotoxic compared to DLD-1 cells, the increase in concentrations of which led to a considerable decrease in cell viability. GO and LPRGO cell viability effects were like each other because they both had around 75% cell viability at concentrations above 100 μg/mL, with cell viability increasing up to 90% below 100 μg/mL concentrations. ERGO and LLRGO behaved in similar ways when interacting with CHO cells. They both have around 70% cell viability up to 50 μg/mL concentration, and they have 80% cell viability at 25 μg/mL concentration and below. These viability values for CHO cell lines were lower than the values obtained for DLD-1 cells. It was also determined that CHO cell viability in the presence of ERGO and LLGO was lower than that of GO and LPRGO. From these results, it can be said that there is a concentration-dependent cytotoxic effect on both DLD-1 and CHO cells, which is consistent with results reported in the literature. The main difference between this finding and that of existing literature is the observation of little toxicity even at high concentrations. Around 70–80% cell viability was observed at concentrations of 200 μg/mL for both GO and RGO. Wate et al. examined the cytotoxicity of GO nano systems on MCF-1 breast cancer cell lines and reported that their cell viability assay revealed no indication of cytotoxicity even at a concentration of 100 μg/mL.^68^ These high concentration values were found to be compatible with our results.

In the literature, it was reported that cell membranes can be penetrated by single- and few-layered graphene having sharp edges, resulting in membrane disruption and cytoplasmic material leakage. The major cytotoxicity responses to GO and RGO when exposed to different cell lines are DNA damage, cell cycle arrest, and oxidative stresses within the cell, which are possibly due to the generation of reactive oxygen species and the deregulation of antioxidant genes.^69^ Most research to date has focused primarily on the toxicity caused by pristine graphene and GO, but it has not been fully recognized that RGO is biocompatible. For biological uses, such as drug delivery carriers, diagnostic sensors, biomarkers, and antimicrobial agents, RGO has recently been evaluated.^33^ However, some in vitro adverse effects have been shown to exist, including the development of reactive oxygen species, cell apoptosis, inflammatory cytokines, membrane integrity depletion, membrane distress caused by direct interaction with sharp edges of RGO, and inflammatory infiltration of cells.^34^ Recent studies have also shown that RGO is likely to be toxic and may concentration-dependently incorporate cell membranes and induce programmed cell death, especially at concentrations greater than 50 μg/L.^35,36,70^ To overcome these concerns and enhance the bioavailability of RGOs, it is important to examine their effect on the safety of living systems and to learn the importance of the toxicological processes that will improve the preparation of RGOs using current methods.

By blocking the immune tolerance of the host cells to employ blood vessel factories for their survival, RGO-mediated toxicity theoretically triggers the poor supply of vital nutrients to cancer cells. One of the main paradigms contributing to graphene toxicology is oxidative stress, which decreases cell viability and also hinders the absorption of important proteins and nutrients into cells.^35^ The development and abolition of reactive oxygen species are well-adjusted within the cells, and lipid peroxidation, mitochondrial dysfunction, apoptosis, and necrosis could be caused by changes in balance.^71^ The development of reactive oxygen species to trigger oxidative stress is thought to be a significant cause of graphene nanocomposite toxicity.^71^ In addition, it is shown that cell membrane disturbance, oxidative stresses, and the close interaction of the sharp edges with the cells are assumed to be mainly responsible for the toxicity of RGO, based on the current literature research.

This study also demonstrated whether the effects of GO, ERGO, LLRGO, and LPRGO induce toxicity through apoptosis or necrosis in both DLD-1 and CHO cells, as seen in Tables 4 and 5. They showed very low apoptotic and necrotic responses. When we compared these low values between apoptosis and necrosis, the molecular mechanism of cell death was necrosis. In CHO cells, necrosis values were higher than the apoptosis indexes. The difference between apoptosis and necrosis index for CHO cells was larger when compared to DLD-1 cell lines. There was a decrease in the apoptosis/necrosis index with the decrease in concentration values.

**Table 4:** Apoptotic/Necrotic Index Results of Samples Applied to DLD-1 Cells

concentration (μg/mL)	GO		ERGO		LLRGO		LPRGO	
	%apoptisis	%necrosis	%apoptisis	%necrosis	%apoptisis	%necrosis	%apoptisis	%necrosis
100	1.38 ± 0.4	3.67 ± 1.2	4.21 ± 1.2	8.42 ± 2.4	2.33 ± 0.6	3.10 ± 1.6	2.62 ± 0.6	5.76 ± 1.7
25	1.46 ± 0.6	2.19 ± 1.7	2.86 ± 0.9	4.76 ± 1.8	0.79 ± 0.9	3.97 ± 1.7	1.27 ± 0.5	2.53 ± 1.4

**Table 5:** Apoptotic/Necrotic Index Results of Samples Applied to CHO Cells

concentration (μg/mL)	GO		ERGO		LLRGO		LPRGO	
	%apoptisis	%necrosis	%apoptisis	%necrosis	%apoptisis	%necrosis	%apoptisis	%necrosis
100	4.44 ± 2.4	12.22 ± 4.2	6.88 ± 1.2	11.93 ± 3.3	4.01 ± 1.6	10.03 ± 2.8	3.83 ± 1.4	8.13 ± 2.6
25	2.05 ± 0.6	6.15 ± 0.9	4.88 ± 1.5	9.76 ± 2.6	3.23 ± 1.3	6.91 ± 1.7	1.33 ± 0.2	3.33 ± 0.8

**Figure 6:**
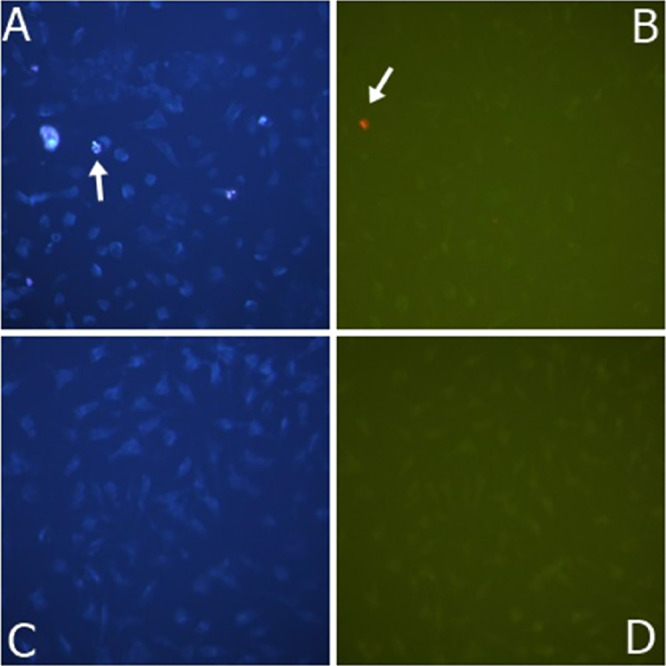
Apoptotic/necrotic photographs of GO sample of DLD-1 cells with a concentration of 100 μg/mL. (A) GO apoptotic result of 100 μg/mL; (B) GO, 100 μg/mL necrotic result; (C) control group apoptotic result; and (D) control group necrotic result.

In Figure 6, photographs of apoptosis and necrosis of DLD-1 cells in different concentrations of GO samples were given. The arrows show some of the apoptotic cells. The apoptosis and necrosis cell nuclei were bright and fragmented, while those that did not undergo apoptosis appeared pale blue. Photographs were taken at 200× magnification with a Leica inverted fluorescent microscope.

**Figure 7:**
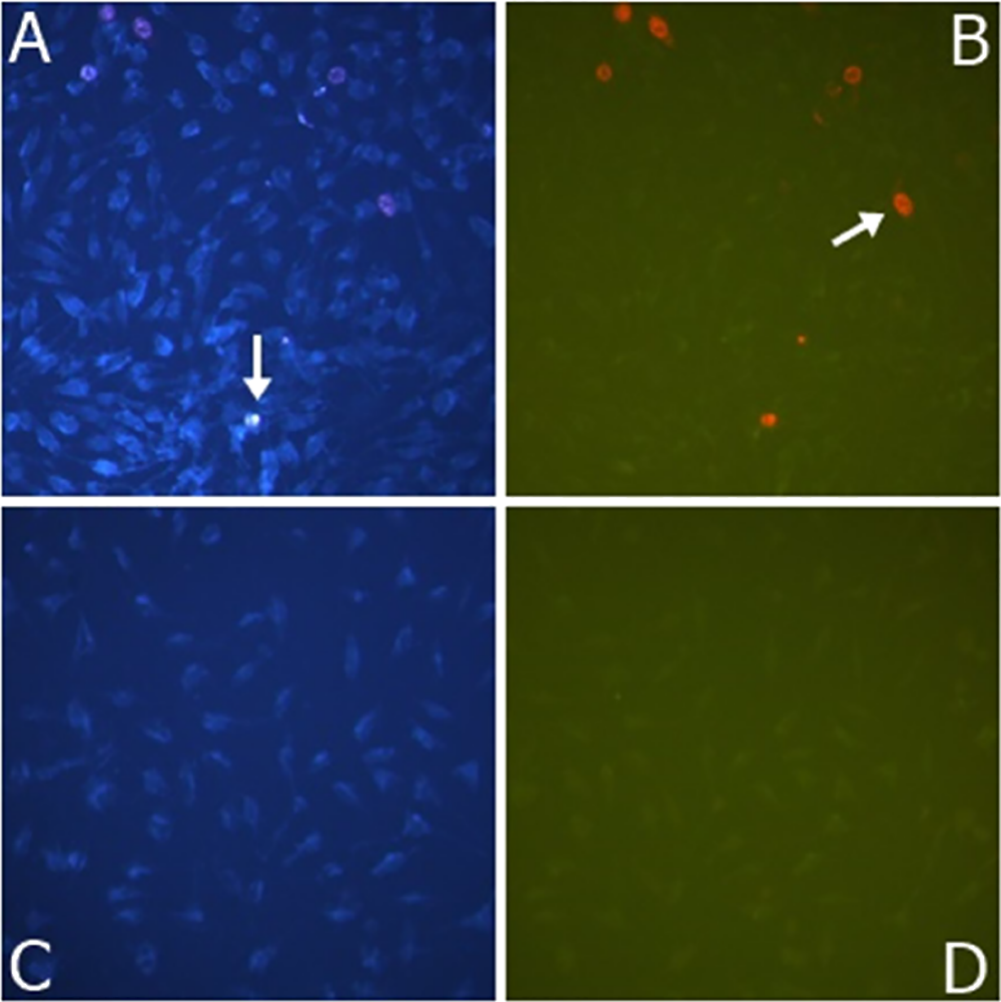
Apoptotic/necrotic photographs of the LLRGO sample of CHO cells with a concentration of 100 μg/mL. (A) LLRGO, 100 μg/mL apoptotic result; (B) LLRGO, 100 μg/mL necrotic result; (C) control group apoptotic result; and (D) control group necrotic result.

As shown in Figure 7, photographs of apoptosis and necrosis of CHO cells in different concentrations of LLRGO samples were given. The apoptotic cells were shown with an arrow. The apoptosis and necrosis cell nuclei were bright and fragmented, while those that did not undergo apoptosis appeared pale blue. Photographs were taken at 200× magnification with a Leica inverted fluorescent microscope.

**Figure 8:**
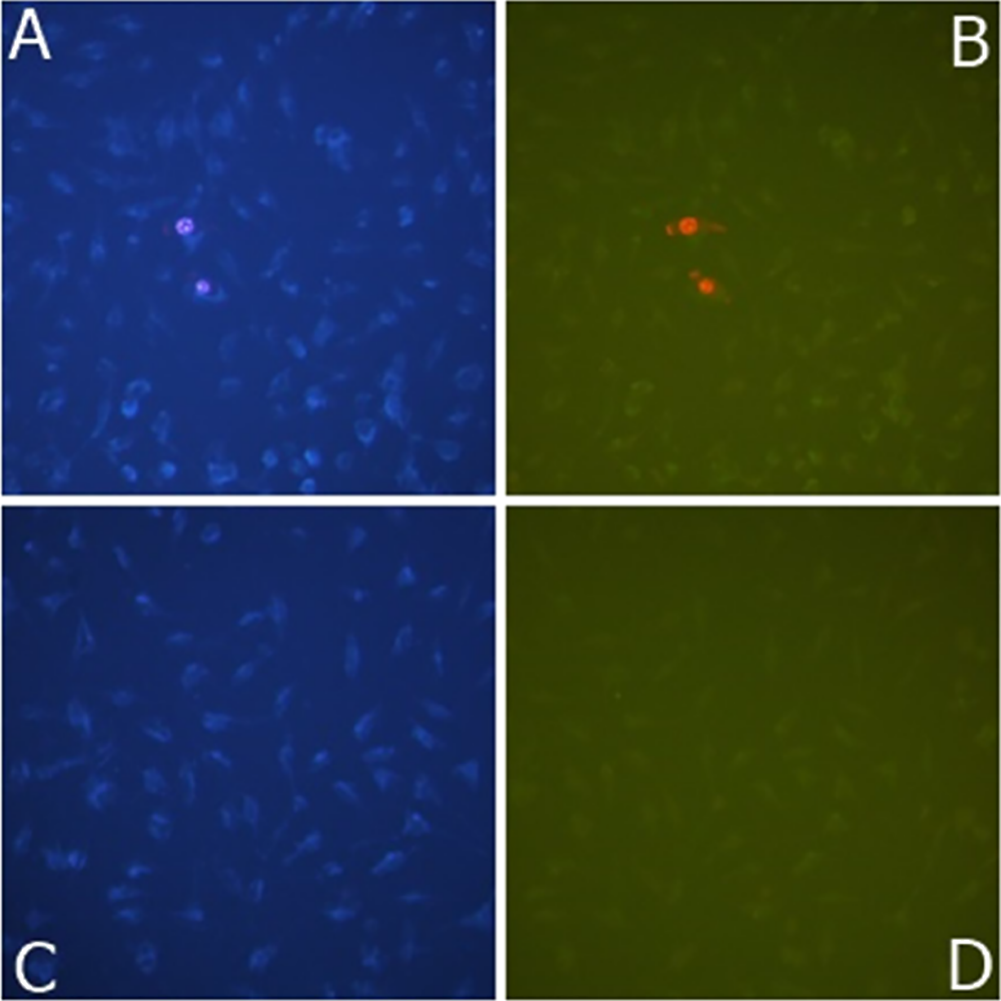
Apoptotic/necrotic photographs of LPRGO sample of CHO cells with a concentration of 100 μg/mL. (A) LPRGO, 100 μg/mL apoptotic result; (B) LPRGO, 100 μg/mL necrotic result; (C) control group apoptotic result (D); and control group necrotic result.

As shown in Figure 8, photographs of apoptosis and necrosis of CHO cells in different concentrations of LPRGO samples were given. The arrows showed some of the apoptotic cells. The apoptosis and necrosis cell nuclei were bright and fragmented, while those that did not undergo apoptosis appeared pale blue. Photographs were taken at 200× magnification with a Leica inverted fluorescent microscope.

## Conclusions

A promising path with many potential uses is the use of various microorganisms to produce reduced graphene oxide (RGO). However, for RGO to be successfully used, it is crucial to comprehend its toxicity and use it safely. Graphene oxide (GO) can be reduced to RGO via microbial reduction techniques, which take advantage of the distinctive enzymatic properties of particular bacterial strains. The capacity to synthesize RGO in bacteria has been studied in *Schewanella* and *Bacillus subtilis*. The characteristics of the resulting RGO can be tailored by selecting the right bacterial strain, allowing for adjustments to the size, surface chemistry, and degree of reduction. The use of bacteria for RGO generation is consistent with sustainable practices because they can utilize renewable carbon sources and function in mild reaction conditions. This method helps to create a production process that is environmentally friendly by reducing the use of harsh chemicals and energy-intensive processes. This research has shown that different microorganisms may successfully reduce graphene oxide in a straightforward and effective manner. The preparation of RGO samples from chemically derived GO was successfully accomplished using three different microorganisms: *E. coli*, *L. lactis*, and *L. plantarum*. The obtained RGOs were characterized, and each was shown to exhibit different physicochemical characteristics depending on the respiratory mechanism of the microorganisms used. They all successfully increased the C/O ratio of RGOs compared to GO by reducing oxygen functional groups. Experiments showed that *L. lactis* reduction was more effective and produced a higher C/O ratio compared to the other microorganisms.

It was shown that the three microorganisms reduced oxygen functional groups and separated the layers effectively. Although the interaction of the materials with DLD-1 and CHO cell lines showed concentration-dependent toxicity, very low toxicity and an apoptotic or necrotic response were generally observed. The results showed that RGOs can be microbially prepared to exhibit desirable properties by changing the microorganisms used in the reduction process, the product of which can be used for various health applications. Their low toxicity and apoptotic/necrotic responses further enhance their continuous study and use in various bioengineering applications.

## References

[ref1] Novoselov K. S.; Geim A. K.; Morozov S. V.; Jiang D.; Zhang Y.; Dubonos S. V.; Grigorieva I. V.; Firsov A. A. Electric Field Effect in Atomically Thin Carbon Films. Science 2004, 306 (5696), 666–669. 10.1126/science.1102896. 15499015

[ref2] Balandin A. A.; Ghosh S.; Bao W.; Calizo I.; Teweldebrhan D.; Miao F.; Lau C. N. Superior Thermal Conductivity of Single-Layer Graphene. Nano Lett. 2008, 8 (3), 902–907. 10.1021/nl0731872. 18284217

[ref3] Orlita M.; Faugeras C.; Plochocka P.; Neugebauer P.; Martinez G.; Maude D. K.; Barra A.-L.; Sprinkle M.; Berger C.; de Heer W. A. Approaching the Dirac Point in High-Mobility Multilayer Epitaxial Graphene. Phys. Rev. Lett. 2008, 101 (26), 267601 10.1103/PhysRevLett.101.267601. 19437673

[ref4] Eda G.; Chhowalla M. Chemically Derived Graphene Oxide: Towards Large-area Thin-film Electronics and Optoelectronics. Adv. Mater. 2010, 22 (22), 2392–2415. 10.1002/adma.200903689. 20432408

[ref5] Berger C.; Song Z.; Li X.; Wu X.; Brown N.; Naud C.; Mayou D.; Li T.; Hass J.; Marchenkov A. N. Electronic Confinement and Coherence in Patterned Epitaxial Graphene. Science 2006, 312 (5777), 1191–1196. 10.1126/science.1125925. 16614173

[ref6] Wintterlin J.; Bocquet M.-L. Graphene on Metal Surfaces. Surf. Sci. 2009, 603 (10–12), 1841–1852. 10.1016/j.susc.2008.08.037.

[ref7] Kim K. S.; Zhao Y.; Jang H.; Lee S. Y.; Kim J. M.; Kim K. S.; Ahn J.-H.; Kim P.; Choi J.-Y.; Hong B. H. Large-Scale Pattern Growth of Graphene Films for Stretchable Transparent Electrodes. Nature 2009, 457 (7230), 706–710. 10.1038/nature07719. 19145232

[ref8] McAllister M. J.; Li J.-L.; Adamson D. H.; Schniepp H. C.; Abdala A. A.; Liu J.; Herrera-Alonso M.; Milius D. L.; Car R.; Prud’homme R. K. Single Sheet Functionalized Graphene by Oxidation and Thermal Expansion of Graphite. Chem. Mater. 2007, 19 (18), 4396–4404. 10.1021/cm0630800.

[ref9] Ambrosi A.; Pumera M. Precise Tuning of Surface Composition and Electron-transfer Properties of Graphene Oxide Films through Electroreduction. Chem.—Eur. J. 2013, 19 (15), 4748–4753. 10.1002/chem.201204226. 23436748

[ref10] Stankovich S.; Dikin D. A.; Piner R. D.; Kohlhaas K. A.; Kleinhammes A.; Jia Y.; Wu Y.; Nguyen S. T.; Ruoff R. S. Synthesis of Graphene-Based Nanosheets via Chemical Reduction of Exfoliated Graphite Oxide. Carbon N. Y. 2007, 45 (7), 1558–1565. 10.1016/j.carbon.2007.02.034.

[ref11] Park S.; Ruoff R. S. Chemical Methods for the Production of Graphenes. Nat. Nanotechnol. 2009, 4 (4), 217–224. 10.1038/nnano.2009.58. 19350030

[ref12] Wang J.; Salihi E. C.; šiller L. Green Reduction of Graphene Oxide Using Alanine. Mater. Sci. Eng., C 2017, 72, 1–6. 10.1016/j.msec.2016.11.017. 28024564

[ref13] Dubin S.; Gilje S.; Wang K.; Tung V. C.; Cha K.; Hall A. S.; Farrar J.; Varshneya R.; Yang Y.; Kaner R. B. A One-Step, Solvothermal Reduction Method for Producing Reduced Graphene Oxide Dispersions in Organic Solvents. ACS Nano 2010, 4 (7), 3845–3852. 10.1021/nn100511a. 20586422 PMC3939021

[ref14] Sengupta I.; Chakraborty S.; Talukdar M.; Pal S. K.; Chakraborty S. Thermal Reduction of Graphene Oxide: How Temperature Influences Purity. J. Mater. Res. 2018, 33 (23), 4113–4122. 10.1557/jmr.2018.338.

[ref15] Hung Y.-F.; Cheng C.; Huang C.-K.; Yang C.-R. A Facile Method for Batch Preparation of Electrochemically Reduced Graphene Oxide. Nanomaterials 2019, 9 (3), 376 10.3390/nano9030376. 30841616 PMC6473953

[ref16] Wang Y.; Shi Z.; Yin J. Facile Synthesis of Soluble Graphene via a Green Reduction of Graphene Oxide in Tea Solution and Its Biocomposites. ACS Appl. Mater. Interfaces 2011, 3 (4), 1127–1133. 10.1021/am1012613. 21438576

[ref17] Akhavan O.; Ghaderi E.; Aghayee S.; Fereydooni Y.; Talebi A. The Use of a Glucose-Reduced Graphene Oxide Suspension for Photothermal Cancer Therapy. J. Mater. Chem. 2012, 22 (27), 13773–13781. 10.1039/c2jm31396k.

[ref18] Duan G.; Zhang Y.; Luan B.; Weber J. K.; Zhou R. W.; Yang Z.; Zhao L.; Xu J.; Luo J.; Zhou R. Graphene-Induced Pore Formation on Cell Membranes. Sci. Rep. 2017, 7 (1), 42767 10.1038/srep42767. 28218295 PMC5317030

[ref19] Kuila T.; Bose S.; Khanra P.; Mishra A. K.; Kim N. H.; Lee J. H. A Green Approach for the Reduction of Graphene Oxide by Wild Carrot Root. Carbon N. Y. 2012, 50 (3), 914–921. 10.1016/j.carbon.2011.09.053.

[ref20] Salas E. C.; Sun Z.; Lüttge A.; Tour J. M. Reduction of Graphene Oxide via Bacterial Respiration. ACS Nano 2010, 4 (8), 4852–4856. 10.1021/nn101081t. 20731460

[ref21] Zhang J.; Yang H.; Shen G.; Cheng P.; Zhang J.; Guo S. Reduction of Graphene Oxide via L-Ascorbic Acid. Chem. Commun. 2010, 46 (7), 1112–1114. 10.1039/B917705A. 20126730

[ref22] Liu J.; Fu S.; Yuan B.; Li Y.; Deng Z. Toward a Universal “Adhesive Nanosheet” for the Assembly of Multiple Nanoparticles Based on a Protein-Induced Reduction/Decoration of Graphene Oxide. J. Am. Chem. Soc. 2010, 132 (21), 7279–7281. 10.1021/ja100938r. 20462190

[ref23] De Silva K. K. H.; Huang H.-H.; Joshi R. K.; Yoshimura M. Chemical Reduction of Graphene Oxide Using Green Reductants. Carbon N. Y. 2017, 119, 190–199. 10.1016/j.carbon.2017.04.025.

[ref24] Li Y.; Yuan H.; von Dem Bussche A.; Creighton M.; Hurt R. H.; Kane A. B.; Gao H. Graphene Microsheets Enter Cells through Spontaneous Membrane Penetration at Edge Asperities and Corner Sites. Proc. Natl. Acad. Sci. U. S. A. 2013, 110 (30), 12295–12300. 10.1073/pnas.1222276110. 23840061 PMC3725082

[ref25] Raveendran S.; Chauhan N.; Nakajima Y.; Toshiaki H.; Kurosu S.; Tanizawa Y.; Tero R.; Yoshida Y.; Hanajiri T.; Maekawa T. Ecofriendly Route for the Synthesis of Highly Conductive Graphene Using Extremophiles for Green Electronics and Bioscience. Part. Part. Syst. Charact. 2013, 30 (7), 573–578. 10.1002/ppsc.201200126.

[ref26] Kumar S.; Chatterjee K. Comprehensive Review on the Use of Graphene-Based Substrates for Regenerative Medicine and Biomedical Devices. ACS Appl. Mater. Interfaces 2016, 8 (40), 26431–26457. 10.1021/acsami.6b09801. 27662057

[ref27] Wang G.; Qian F.; Saltikov C. W.; Jiao Y.; Li Y. Microbial Reduction of Graphene Oxide by Shewanella. Nano Res. 2011, 4, 563–570. 10.1007/s12274-011-0112-2.

[ref28] Zhang H.; Yu X.; Guo D.; Qu B.; Zhang M.; Li Q.; Wang T. Synthesis of Bacteria Promoted Reduced Graphene Oxide-Nickel Sulfide Networks for Advanced Supercapacitors. ACS Appl. Mater. Interfaces 2013, 5 (15), 7335–7340. 10.1021/am401680m. 23751359

[ref29] Akhavan O.; Ghaderi E. Escherichia Coli Bacteria Reduce Graphene Oxide to Bactericidal Graphene in a Self-Limiting Manner. Carbon N. Y. 2012, 50 (5), 1853–1860. 10.1016/j.carbon.2011.12.035.

[ref30] Schütz B.; Seidel J.; Sturm G.; Einsle O.; Gescher J. Investigation of the Electron Transport Chain to and the Catalytic Activity of the Diheme Cytochrome c Peroxidase CcpA of Shewanella Oneidensis. Appl. Environ. Microbiol. 2011, 77 (17), 6172–6180. 10.1128/AEM.00606-11. 21742904 PMC3165401

[ref31] Utkan G.; Ozturk T.; Duygulu O.; Tahtasakal E.; Denizci A. A. Microbial Reduction of Graphene Oxide By Lactobacillus Plantarum. Int. J. Nanosci. Nanotechnol. 2019, 15 (2), 127–136.

[ref32] Utkan G. Effective Reduction of Graphene Oxide via Lactococcus Lactis. Celal Bayar Univ. J. Sci. 2020, 16 (2), 155–160. 10.18466/cbayarfbe.710338.

[ref33] Zhang X.; Nan X.; Shi W.; Sun Y.; Su H.; He Y.; Liu X.; Zhang Z.; Ge D. Polydopamine-Functionalized Nanographene Oxide: A Versatile Nanocarrier for Chemotherapy and Photothermal Therapy. Nanotechnology 2017, 28 (29), 295102 10.1088/1361-6528/aa761b. 28656906

[ref34] Hu W.; Peng C.; Luo W.; Lv M.; Li X.; Li D.; Huang Q.; Fan C. Graphene-Based Antibacterial Paper. ACS Nano 2010, 4 (7), 4317–4323. 10.1021/nn101097v. 20593851

[ref35] Volkov Y.; McIntyre J.; Prina-Mello A. Graphene Toxicity as a Double-Edged Sword of Risks and Exploitable Opportunities: A Critical Analysis of the Most Recent Trends and Developments. 2D Mater. 2017, 4 (2), 022001 10.1088/2053-1583/aa5476.

[ref36] Seabra A. B.; Paula A. J.; de Lima R.; Alves O. L.; Durán N. Nanotoxicity of Graphene and Graphene Oxide. Chem. Res. Toxicol. 2014, 27 (2), 159–168. 10.1021/tx400385x. 24422439

[ref37] Hummers W. S. Jr; Offeman R. E. Preparation of Graphitic Oxide. J. Am. Chem. Soc. 1958, 80 (6), 1339 10.1021/ja01539a017.

[ref38] Li Z. Q.; Lu C. J.; Xia Z. P.; Zhou Y.; Luo Z. X-Ray Diffraction Patterns of Graphite and Turbostratic Carbon. Carbon N. Y. 2007, 45 (8), 1686–1695. 10.1016/j.carbon.2007.03.038.

[ref39] Feng J.; Ye Y.; Xiao M.; Wu G.; Ke Y. Synthetic Routes of the Reduced Graphene Oxide. Chem. Pap. 2020, 74, 3767–3783. 10.1007/s11696-020-01196-0.

[ref40] Dong L.; Yang J.; Chhowalla M.; Loh K. P. Synthesis and Reduction of Large Sized Graphene Oxide Sheets. Chem. Soc. Rev. 2017, 46 (23), 7306–7316. 10.1039/C7CS00485K. 29051935

[ref41] Ni Z.; Wang Y.; Yu T.; Shen Z. Raman Spectroscopy and Imaging of Graphene. Nano Res. 2008, 1, 273–291. 10.1007/s12274-008-8036-1.

[ref42] Schulte M.; Frick K.; Gnandt E.; Jurkovic S.; Burschel S.; Labatzke R.; Aierstock K.; Fiegen D.; Wohlwend D.; Gerhardt S. A Mechanism to Prevent Production of Reactive Oxygen Species by Escherichia Coli Respiratory Complex I. Nat. Commun. 2019, 10 (1), 2551 10.1038/s41467-019-10429-0. 31186428 PMC6560083

[ref43] Sanders J. W.; Venema G.; Kok J. Environmental Stress Responses in Lactococcus Lactis. FEMS Microbiol. Rev. 1999, 23 (4), 483–501. 10.1111/j.1574-6976.1999.tb00409.x.

[ref44] Brooijmans R. J. W.; De Vos W. M.; Hugenholtz J. Lactobacillus Plantarum WCFS1 Electron Transport Chains. Appl. Environ. Microbiol. 2009, 75 (11), 3580–3585. 10.1128/AEM.00147-09. 19346351 PMC2687314

[ref45] Gholami A.; Emadi F.; Amini A.; Shokripour M.; Chashmpoosh M.; Omidifar N. Functionalization of Graphene Oxide Nanosheets Can Reduce Their Cytotoxicity to Dental Pulp Stem Cells. J. Nanomater. 2020, 2020, 1–14. 10.1155/2020/6942707.

[ref46] Faiz M. S. A.; Azurahanim C. A. C.; Raba’ah S. A.; Ruzniza M. Z. Low Cost and Green Approach in the Reduction of Graphene Oxide (GO) Using Palm Oil Leaves Extract for Potential in Industrial Applications. Results Phys. 2020, 16, 102954 10.1016/j.rinp.2020.102954.

[ref47] Fan Z.; Wang K.; Wei T.; Yan J.; Song L.; Shao B. An Environmentally Friendly and Efficient Route for the Reduction of Graphene Oxide by Aluminum Powder. Carbon N. Y. 2010, 48 (5), 1686–1689. 10.1016/j.carbon.2009.12.063.

[ref48] El Achaby M.; Arrakhiz F. Z.; Vaudreuil S.; Essassi E. M.; Qaiss A. Piezoelectric β-Polymorph Formation and Properties Enhancement in Graphene Oxide–PVDF Nanocomposite Films. Appl. Surf. Sci. 2012, 258 (19), 7668–7677. 10.1016/j.apsusc.2012.04.118.

[ref49] Bagri A.; Mattevi C.; Acik M.; Chabal Y. J.; Chhowalla M.; Shenoy V. B. Structural Evolution during the Reduction of Chemically Derived Graphene Oxide. Nat. Chem. 2010, 2 (7), 581–587. 10.1038/nchem.686. 20571578

[ref50] Sanchez V. C.; Jachak A.; Hurt R. H.; Kane A. B. Biological Interactions of Graphene-Family Nanomaterials: An Interdisciplinary Review. Chem. Res. Toxicol. 2012, 25 (1), 15–34. 10.1021/tx200339h. 21954945 PMC3259226

[ref51] Yue H.; Wei W.; Yue Z.; Wang B.; Luo N.; Gao Y.; Ma D.; Ma G.; Su Z. The Role of the Lateral Dimension of Graphene Oxide in the Regulation of Cellular Responses. Biomaterials 2012, 33 (16), 4013–4021. 10.1016/j.biomaterials.2012.02.021. 22381473

[ref52] Mendes R. G.; Koch B.; Bachmatiuk A.; Ma X.; Sanchez S.; Damm C.; Schmidt O. G.; Gemming T.; Eckert J.; Rümmeli M. H. A Size Dependent Evaluation of the Cytotoxicity and Uptake of Nanographene Oxide. J. Mater. Chem. B 2015, 3 (12), 2522–2529. 10.1039/C5TB00180C. 32262127

[ref53] Zhang W.; Yan L.; Li M.; Zhao R.; Yang X.; Ji T.; Gu Z.; Yin J.-J.; Gao X.; Nie G. Deciphering the Underlying Mechanisms of Oxidation-State Dependent Cytotoxicity of Graphene Oxide on Mammalian Cells. Toxicol. Lett. 2015, 237 (2), 61–71. 10.1016/j.toxlet.2015.05.021. 26047786

[ref54] Wu S.-Y.; An S. S. A.; Hulme J. Current Applications of Graphene Oxide in Nanomedicine. Int. J. Nanomedicine 2015, 10 (sup1), 9–24. 10.2147/IJN.S88285. 26345988 PMC4554423

[ref55] Zhang B.; Wei P.; Zhou Z.; Wei T. Interactions of Graphene with Mammalian Cells: Molecular Mechanisms and Biomedical Insights. Adv. Drug Delivery Rev. 2016, 105, 145–162. 10.1016/j.addr.2016.08.009. 27569910

[ref56] Gurunathan S.; Han J. W.; Dayem A. A.; Eppakayala V.; Kim J.-H. Oxidative Stress-Mediated Antibacterial Activity of Graphene Oxide and Reduced Graphene Oxide in Pseudomonas Aeruginosa. Int. J. Nanomedicine 2012, 7, 5901–5914. 10.2147/IJN.S37397. 23226696 PMC3514835

[ref57] Gurunathan S.; Kim J.-H. Graphene Oxide–Silver Nanoparticles Nanocomposite Stimulates Differentiation in Human Neuroblastoma Cancer Cells (SH-SY5Y). Int. J. Mol. Sci. 2017, 18 (12), 2549 10.3390/ijms18122549. 29182571 PMC5751152

[ref58] Chatterjee N.; Eom H.-J.; Choi J. A Systems Toxicology Approach to the Surface Functionality Control of Graphene–Cell Interactions. Biomaterials 2014, 35 (4), 1109–1127. 10.1016/j.biomaterials.2013.09.108. 24211078

[ref59] Jaworski S.; Sawosz E.; Kutwin M.; Wierzbicki M.; Hinzmann M.; Grodzik M.; Winnicka A.; Lipińska L.; Włodyga K.; Chwalibog A. In Vitro and in Vivo Effects of Graphene Oxide and Reduced Graphene Oxide on Glioblastoma. Int. J. Nanomedicine 2015, 10, 1585–1596. 10.2147/IJN.S77591. 25759581 PMC4346365

[ref60] Chang Y.; Yang S.-T.; Liu J.-H.; Dong E.; Wang Y.; Cao A.; Liu Y.; Wang H. In Vitro Toxicity Evaluation of Graphene Oxide on A549 Cells. Toxicol. Lett. 2011, 200 (3), 201–210. 10.1016/j.toxlet.2010.11.016. 21130147

[ref61] Hu W.; Peng C.; Lv M.; Li X.; Zhang Y.; Chen N.; Fan C.; Huang Q. Protein Corona-Mediated Mitigation of Cytotoxicity of Graphene Oxide. ACS Nano 2011, 5 (5), 3693–3700. 10.1021/nn200021j. 21500856

[ref62] Wang K.; Ruan J.; Song H.; Zhang J.; Wo Y.; Guo S.; Cui D. Biocompatibility of Graphene Oxide. Nanoscale Res. Lett. 2011, 6 (1), 8 10.1007/s11671-010-9751-6. 27502632 PMC3212228

[ref63] Lammel T.; Boisseaux P.; Fernández-Cruz M.-L.; Navas J. M. Internalization and Cytotoxicity of Graphene Oxide and Carboxyl Graphene Nanoplatelets in the Human Hepatocellular Carcinoma Cell Line Hep G2. Part. Fibre Toxicol. 2013, 10, 27 10.1186/1743-8977-10-27. 23849434 PMC3734190

[ref64] Pelin M.; Fusco L.; Martín C.; Sosa S.; Frontiñán-Rubio J.; Gonzaĭlez-Domínguez J. M.; Durán-Prado M.; Vázquez E.; Prato M.; Tubaro A. Graphene and Graphene Oxide Induce ROS Production in Human HaCaT Skin Keratinocytes: The Role of Xanthine Oxidase and NADH Dehydrogenase. Nanoscale 2018, 10 (25), 11820–11830. 10.1039/C8NR02933D. 29920573

[ref65] Zhang Y.; Ali S. F.; Dervishi E.; Xu Y.; Li Z.; Casciano D.; Biris A. S. Cytotoxicity Effects of Graphene and Single-Wall Carbon Nanotubes in Neural Phaeochromocytoma-Derived PC12 Cells. ACS Nano 2010, 4 (6), 3181–3186. 10.1021/nn1007176. 20481456

[ref66] Akhavan O.; Ghaderi E.; Akhavan A. Size-Dependent Genotoxicity of Graphene Nanoplatelets in Human Stem Cells. Biomaterials 2012, 33 (32), 8017–8025. 10.1016/j.biomaterials.2012.07.040. 22863381

[ref67] Girase B.; Shah J. S.; Misra R. D. K. Cellular Mechanics of Modulated Osteoblasts Functions in Graphene Oxide Reinforced Elastomers. Adv. Eng. Mater. 2012, 14 (4), B101–B111. 10.1002/adem.201180028.

[ref68] Wate P. S.; Banerjee S. S.; Jalota-Badhwar A.; Mascarenhas R. R.; Zope K. R.; Khandare J.; Misra R. D. K. Cellular Imaging Using Biocompatible Dendrimer-Functionalized Graphene Oxide-Based Fluorescent Probe Anchored with Magnetic Nanoparticles. Nanotechnology 2012, 23 (41), 415101 10.1088/0957-4484/23/41/415101. 23010805

[ref69] Syama S.; Aby C. P.; Maekawa T.; Sakthikumar D.; Mohanan P. V. Nano-Bio Compatibility of PEGylated Reduced Graphene Oxide on Mesenchymal Stem Cells. 2D Mater. 2017, 4 (2), 025066 10.1088/2053-1583/aa65c2.

[ref70] Mittal S.; Kumar V.; Dhiman N.; Chauhan L. K. S.; Pasricha R.; Pandey A. K. Physico-Chemical Properties Based Differential Toxicity of Graphene Oxide/Reduced Graphene Oxide in Human Lung Cells Mediated through Oxidative Stress. Sci. Rep. 2016, 6 (1), 39548 10.1038/srep39548. 28000740 PMC5175188

[ref71] Palmieri V.; Lauriola M. C.; Ciasca G.; Conti C.; De Spirito M.; Papi M. The Graphene Oxide Contradictory Effects against Human Pathogens. Nanotechnology 2017, 28 (15), 152001 10.1088/1361-6528/aa6150. 28303804

